# GmTGA9-GmDYT1 Regulates Anther Wall Development to Affect Male Fertility in Soybean

**DOI:** 10.3390/plants15101510

**Published:** 2026-05-15

**Authors:** Shuo Wu, Wanqing Du, Kexuan Liu, Zhenggang Wang, Yujuan Gu, Xianlong Ding, Shouping Yang

**Affiliations:** 1 National Innovation Platform for Soybean Breeding and Industry-Education Integration, Nanjing 210095, China; 2 Zhongshan Biological Breeding Laboratory (ZSBBL), Nanjing 210095, China; 3 Key Laboratory of Biology and Genetics and Breeding for Soybean, Ministry of Agriculture and Rural Affairs of the People’s Republic of China, Nanjing 210095, China; 4 Jiangsu Key Laboratory of Soybean Biotechnology and Intelligent Breeding, Nanjing 210095, China; 5 State Key Laboratory of Crop Genetics & Germplasm Enhancement and Utilization, Nanjing 210095, China; 6 Jiangsu Collaborative Innovation Center for Modern Crop Production, Nanjing 210095, China; 7 College of Agriculture, Nanjing Agricultural University, Nanjing 210095, China

**Keywords:** soybean (*Glycine max* (L.) Merr.), *GmTGA9d*, *GmDYT1*, male fertility, anther wall development

## Abstract

The scarcity of nuclear male-sterile (NMS) lines severely constrains the development of hybrid breeding in soybean. This study highlights the important role of a conserved GmTGA9-GmDYT1 regulatory module in controlling soybean anther development and male fertility. Through CRISPR/Cas9-mediated screening of the four *TGA9* genes in soybean, we discovered that *GmTGA9c* and *GmTGA9d* may be involved in male fertility. The *tga9d* single-gene mutation caused abnormal thickening of the anther middle layer and impeded anther dehiscence, resulting in partial male sterility. We then found that GmTGA9d directly bound to and inhibited its downstream target gene, *GmDYT1c.* Furthermore, genetic evidence supports that *GmDYT1a* and *GmDYT1c* have functionally overlapping roles. Mutation of both genes caused aberrant degradation of the anther middle layer and tapetum, interrupting microspore development and resulting in complete male sterility. This study provides evidence that the GmTGA9-GmDYT1 pathway in soybean plays important roles in regulating male sterility by controlling the development of the anther wall. These findings provide novel mechanistic insights for research on and application of NMS materials in soybean heterosis utilization and hybrid breeding.

## 1. Introduction

Soybean (*Glycine max* (L.) Merr.) is a vital multifunctional crop cultivated for food, feed and oil which is widely grown across the world. It is critical to guarantee global food security and support the development of the livestock industry [1]. Therefore, increasing soybean yield is a major research objective worldwide. The utilization of heterosis has significantly increased crop yields [2]. In soybean, the “three-line” hybrid breeding approach based on cytoplasmic–nuclear interaction male-sterile lines has experienced significant research progress [3,4,5]. However, due to its inherent limitations, the “three-line” technique has not yet gained widespread adoption. As an alternative, nuclear male sterility (NMS), which is governed by nuclear genes, is a widespread phenomenon in plants and forms the basis of other breeding systems. The application of photoperiod-sensitive and thermo-sensitive male sterility technologies has facilitated hybrid breeding, leading to significant yield increases in crops such as rice and wheat [6,7]. New heterosis methods based on NMS have achieved significant progress in other crops in recent years. For example, recessive NMS technology systems developed by biotechnology, primarily seed production technology (SPT) and multi-control sterility (MCS) systems, have been utilized in various crops [8,9,10]. Furthermore, researchers have also proposed that the application of dominant NMS technology holds promise for enhancing crop grain yield in the future of heterosis utilization [11]. However, investigation into utilizing NMS to increase soybean production remains limited. Researchers have proposed that large-scale production of hybrid seeds can be achieved by the barnase/barstar system, offering valuable resources for the study of heterosis in soybean [12]. However, the genetic resources of utilizable NMS genes discovered so far are insufficient; just five NMS genes (*MS1* [13,14,15], *MS2* [16], *MS3* [17], *MS4* [18] and *MS6* [19]) have been cloned in soybean. Thus, acquiring more stable NMS genes is necessary to enhance soybean production through the application of genic male sterility technology.

Abnormalities of the anther often lead to male sterility in various plants. The anther wall is essential to the maturation and growth of pollen grains. From inner to outer, the anther wall is made up of the tapetum, middle layer, endothecium and epidermis [20]. Among these, the middle layer and tapetum play key roles in anther and pollen development. As the pollen matures, prompt degradation of the anther middle layer facilitates anther dehiscence, which indirectly affects pollen release and eventual maturation [21]. Abnormal degradation of the middle layer of anthers leading to male sterility has been reported in other crops. In rice, mutation in the UDP-glucose 4-epimerase (UGE) enzyme led to the abnormal thickening of the middle layer and tapetum, ultimately resulting in male sterility [22]. In maize, loss of function of the *ZmMs33* gene led to ectopic, premature and excessive secondary cell wall thickening in the anther middle layer cells, which restricted cell elongation and impeded nutrient transport between different cell layers of the anther wall [23,24]. The development of the middle layer is closely associated with that of the innermost tapetum, which is crucial for the development of the pollen wall and provides necessary nutrients for the growth of the pollen grains [25]. A conserved core pathway regulating tapetum development has been characterized in rice and *Arabidopsis*, which is “*AtDYT1/OsUDT1-AtTDF1/OsTDF1-AtAMS/OsTDR-AtMYB80/OsMYB80-AtMS1/OsPTC1*” [26,27,28,29,30]. A similar pathway has been identified (*ZmMS32-ZmMS9-ZmbHLH51-ZmMYB84-ZmMS7*) to regulate tapetum and pollen grain development in maize [31,32]. Among the five nuclear male sterility genes characterized in soybean, *MS6*, *MS2* and *MS3* correspond precisely to the homologues of *TDF*, *AMS* and *MS1*, respectively, implying that the essential tapetum regulatory pathway is likely conserved in soybean [16,17,19]. Within this regulatory pathway, DYT1 acts as an upstream master switch and plays important roles in tapetum development. In *Arabidopsis*, a *dyt1* mutant showed abnormally enlarged vacuoles in the inner layers of anthers, resulting in pollen abortion at an early stage of anther development [27]. A mutation in the *UDT1* gene of rice led to a disruption in the differentiation and vacuolation processes of the inner anther layer during meiosis, which inhibited the degradation of the middle layer and failed to produce pollen in the anther locules [33]. And mutations in *DYT1* orthologs led to similar defects in tapetum development in both maize and tomato [34,35]. Overall, DYT1 is involved in regulating the development of the anther wall in flowering plants.

TGACG-binding (TGA) transcription factor is an important and evolutionarily conserved family in plants, playing key roles in regulating stress responses and growth of plants [36]. Specifically, TGA9 and TGA10 have been confirmed to regulate anther development in *Arabidopsis.* Double-mutant *tga9tga10* disrupted abaxial and adaxial development, resulting in the abnormal persistence of the middle layer at maturity and leading to male sterility [37]. In rice, mutation of *TGA10* affected the development of both the endothecium and tapetum, resulting in complete male sterility [38,39]. In maize, complete male sterility required the simultaneous mutation of all three homologous genes of *TGA9*, whereas single or double mutants remained fertile, indicating functional redundancy among these genes. Additionally, mutation of *TGA10* did not affect pollen development, but rather impaired anther dehiscence, leading to male sterility in maize [31]. These findings underscored the pivotal and conserved roles of TGA9 and TGA10 in anther development across species. In summary, TGA transcription factors play crucial roles in regulating anther structure and pollen development in flowering plants. To date, 25 *TGA* genes have been identified in the soybean genome [40]. Researchers have found that TGA transcription factors could regulate soybean responses to salt and drought stress [41,42]. However, studies on the regulation of soybean anther development by TGA transcription factors have remained limited.

Our study investigates whether mutation of *GmTGA9d* affects anther middle layer development and anther dehiscence, thereby contributing to partial male sterility. We also explored whether GmTGA9d, as a transcription factor, can bind to the *GmDYT1c* promoter and suppress its activity. Moreover, we examined the potential roles of *GmDYT1* in anther middle layer and tapetum development. Combining phenotypic analyses of a *dyt1a1c* mutant, we explored the functional association of *GmDYT1a* and *GmDYT1c* in the regulation of anther wall development. Overall, this study aims to provide evidence for a GmTGA9-GmDYT1 module involved in soybean male fertility by regulating the development of the anther middle layer and tapetum, thereby providing a basis for further investigation of the regulatory network of anther and pollen development in soybean.

## 2. Results

### 2.1. Identification of Four Highly Homologous GmTGA9 Genes in Soybean

**Figure 1:**
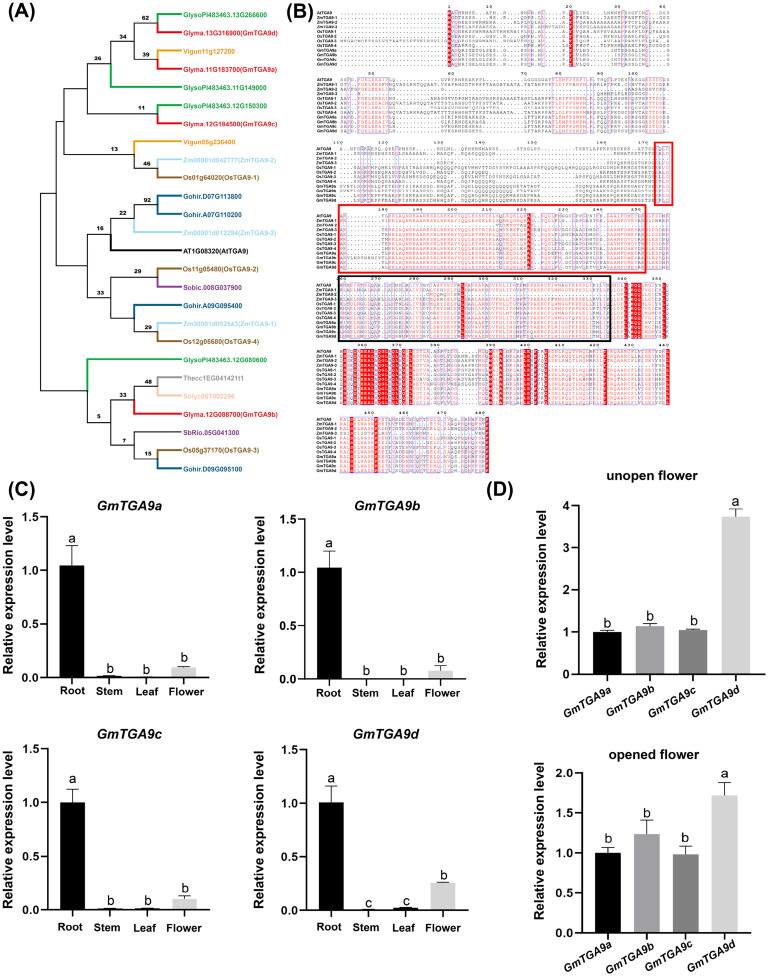
Identification of *TGA9* homologous genes in soybean. (**A**) Phylogenetic tree analysis of TGA9 in multiple plant species, including *Arabidopsis thaliana*, *Glycine soja*, *Vigna unguiculata*, *Solanum lycopersicum*, *Theobroma cacao*, *Zea mays*, *Oryza sativa*, *Sorghum bicolor*, *Gossypium hirsutum* and *Glycine max.* The tree was constructed by MEGA11.0 using the neighbor-joining (NJ) method with 1000 bootstrap replicates based on the amino acid sequences of the TGA9 proteins. (**B**) Protein sequence alignment of four *GmTGA9* genes and their homologs in *Arabidopsis thaliana*, *Oryza sativa* and *Zea mays*. Red-box region indicates the BRLZ domain, and black-box region indicates the DOG1 domain. (**C**) Relative expression level of *GmTGA9a*, *GmTGA9b*, *GmTGA9c* and *GmTGA9d* in root, stem, leaf and flower of Williams 82 (W82). (**D**) Relative expression level of *GmTGA9a*, *GmTGA9b*, *GmTGA9c* and *GmTGA9d* in unopen flowers and opened flowers of W82. Data for each tissue are collected with three biological replicates (*n* = 3), and each biological replicate consists of tissue samples from a single independent plant. For qRT-PCR analysis, each biological sample is examined in three technical replicates. The 2^−ΔΔCT^ method is used to calculate the relative expression levels. Data are presented as the mean ± standard deviation (SD). Values with different letters indicate statistical differences (one-way ANOVA, Duncan’s test, *p*  <  0.05).

We identified four *TGA9* homologous genes in the soybean genome using the AtTGA9 protein sequence, designated as *GmTGA9a*, *GmTGA9b*, *GmTGA9c* and *GmTGA9d*. Their amino acid sequences shared 65.6%, 67.0%, 68.5% and 67.1% identity with AtTGA9, respectively. Multiple sequence alignment revealed extensive conservation within the functional domains of the four soybean *GmTGA9* genes, the three maize *TGA9* sterility genes (*ZmTGA9-1*, *ZmTGA9-2*, *ZmTGA9-3*) and *AtTGA9* (Figure 1B), suggesting functional conservation of TGA9 across species. We constructed a phylogenetic tree containing these four soybean genes and other plant *TGA9* homologs to elucidate their evolutionary relationships. Analysis showed that *GmTGA9a*, *GmTGA9c* and *GmTGA9d* were more closely related to *TGA9* genes from legumes (*Glycine soja* and *Vigna unguiculata*), whereas *GmTGA9b* clustered with those from *Solanum lycopersicum* and *Theobroma cacao*, suggesting possible functional divergence among the four *GmTGA9* genes (Figure 1A). Analysis of tissue-specific expression patterns showed that the expression levels of the four genes were highest in roots, moderate in flowers and nearly undetectable in stems and leaves (Figure 1C). Notably, *GmTGA9d* expression was markedly higher in flowers compared to the other three genes, especially in unopened buds, which suggests its potential involvement in flower bud development (Figure 1D).

### 2.2. Observation of Male Fertility in T_0_ Heterozygous Mutants of GmTGA9

**Figure 2:**
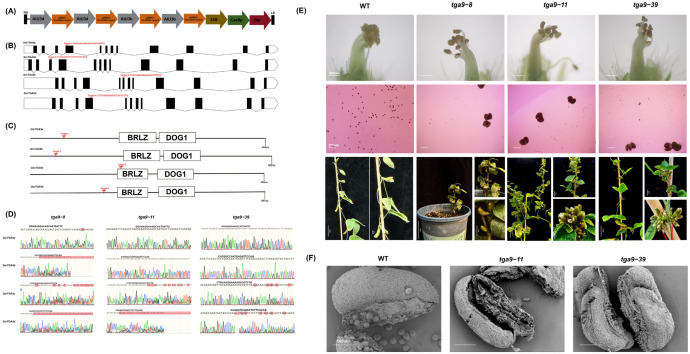
Generation and phenotypic characterization of *tga9* multiple mutants. (**A**) Schematic diagram of the CRISPR-Cas9 gene-editing vector for *GmTGA9a/b/c/d*. (**B**) Schematic of the target sites in *GmTGA9a/b/c/d*. The white rectangle represents the 5’UTR, the white pentagon represents the 3’UTR, black boxes represent exons, and the connecting lines represent introns. The red text above the gene structure indicates the target sequences. (**C**) Schematic of the target sites within the amino acid sequences of *GmTGA9a/b/c/d*. The black line represents the amino acid sequence, and the functional domains BRLZ and DOG1 are indicated within the white boxes. (**D**) Sequencing chromatograms of the target sites in *GmTGA9a/b/c/d* for *tga9-8*, *tga9-11* and *tga9-39*. The target sequences are indicated in black text above the chromatograms. (**E**) Phenotypic analysis of the wild type (WT), *tga9-8*, *tga9-11* and *tga9-39*. The four upper panels show the anther dehiscence phenotype of the mutants. Bar = 500 μm. The four middle panels represent the analysis of pollen viability by iodide (I_2_-KI)-stained pollen grains. Bar = 200 μm. The four lower panels display the phenotypic analysis of the mutants at the mature stage. The scale bars from left to right are 10 cm, 2 cm and 1 cm. (**F**) Comparative scanning electron microscopy (SEM) analysis of anthers from WT, *tga9-11* and *tga9-39*. Bar = 100 μm.

Using the CRISPR-Cas9 system, we designed targets upstream of the conserved domains in the four *GmTGA9* genes (Figure 2A–C) and successfully obtained five T_0_-generation heterozygous mutant plants (*tga9-8, tga9-11, tga9-33, tga9-39* and *tga9-59*). Among them, *tga9-8*, *tga9-11* and *tga9-39* exhibited complete male sterility, characterized by non-dehiscent anthers and no observable fertile pollen grains in the iodine staining (I_2_-KI) assay, which was further confirmed by scanning electron microscopy (SEM) showing shrunken anthers devoid of pollen grains internally (Figure 2E,F).

We conducted sequencing analysis of T_0_ mutant plants to identify candidate genes that may participate in the regulation of soybean male fertility. Specifically, *tga9-8* contained heterozygous edits in *GmTGA9a*, *GmTGA9b*, *GmTGA9c* and *GmTGA9d*, *tga9-11* in *GmTGA9a*, *GmTGA9c* and *GmTGA9d*, and *tga9-39* in *GmTGA9c* and *GmTGA9d* (Table 1, Figure 2D). These three mutants carried heterozygous edits in both *GmTGA9c* and *GmTGA9d*. And T_0_-generation plants of *tga9-33* (with heterozygous edits in *GmTGA9b* and *GmTGA9d*) and *tga9-59* (with heterozygous edits in *GmTGA9a* and *GmTGA9d*) remained fertile (Figure S1A). Through selfing to obtain homozygous progeny, the homozygous *tga9-59* line displayed partial pollen sterility, consistent with the phenotype of the *tga9d* single mutant described in the following result (Figure S1B,D), whereas the homozygous *tga9-33* line was completely fertile (Figure S1D). It is noteworthy that the mutation in *GmTGA9d* in *tga9-33* was a 9 bp deletion that did not cause a frameshift, which may explain the maintained fertility in the homozygous background (Figure S1C). In summary, phenotypic analysis of T_0_ plants served as a preliminary screen, which suggested that *GmTGA9c* and *GmTGA9d* might be involved in regulating soybean male fertility.

### 2.3. Tga9d Single Mutant Exhibits Partial Male Sterility Due to Aberrant Anther Wall Development

**Figure 3:**
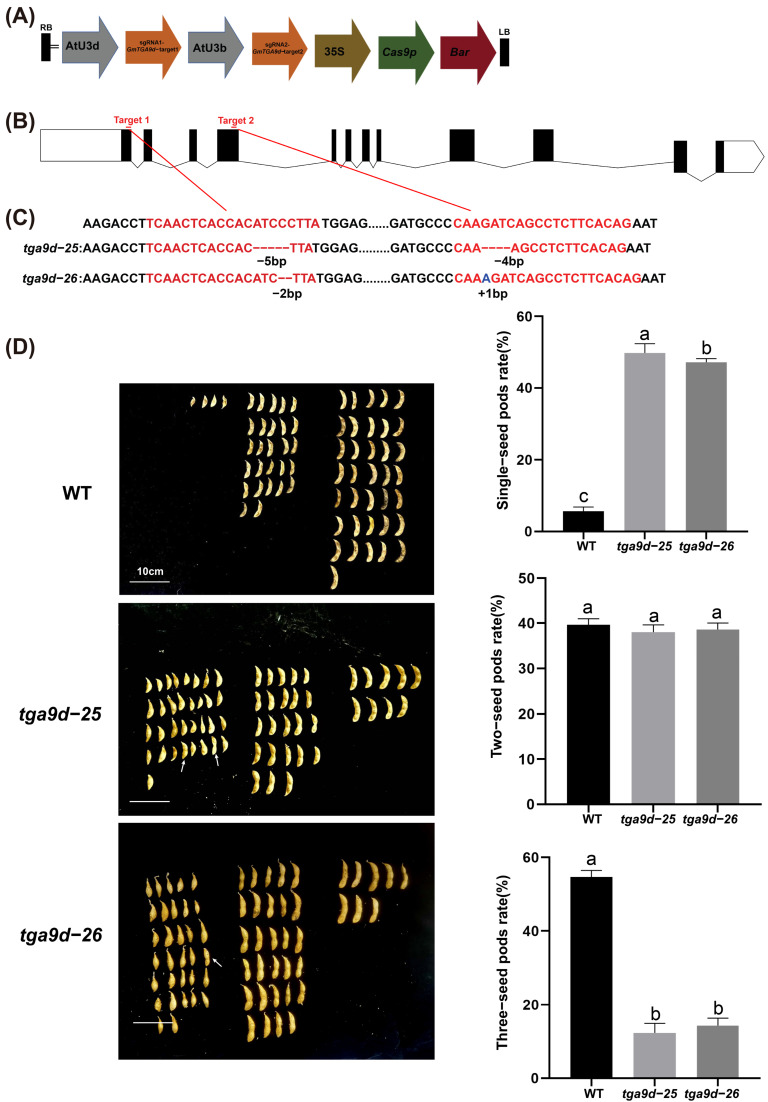
Generation and phenotypic analysis of the two *tga9d* mutants. (**A**) Schematic diagram of the CRISPR-Cas9 vector for *GmTGA9d* single-gene editing. (**B**) Schematic showing the locations of the two target sites within the *GmTGA9d* gene sequence. (**C**) Base editing status at the target sites in the two edited lines, *tga9d-25* and *tga9d-26*. The target sequences are shown in red text. (**D**) Statistical analysis of pod-setting habit in WT, *tga9d-25* and *tga9d-26* at maturity. White arrows indicate two-seeded pods with only one developing seed, which are classified and counted as single-seeded pods. Bar = 10 cm. The three panels on the right show a comparative analysis of the proportions of single-seed, two-seed, and three-seed pods in the WT, *tga9d-25* and *tga9d-26* at maturity. Data are obtained from eight biological replicates (*n* = 8), with statistics collected from eight independently grown plants. Data are presented as the mean ± SD. Values with different letters indicate statistical differences (one-way ANOVA, Duncan’s test, *p*  <  0.05).

To identify the function of *GmTGA9d* in soybean fertility, we created two different editing types of *tga9d* mutants, which were *tga9d-25* and *tga9d-26* (Figure 3A–C). Both mutants underwent homozygous editing at two target sites, resulting in premature termination of translation (Figure S2A,B). The *tga9d-25* and *tga9d-26* mutants exhibited normal vegetative growth, but showed altered pod-setting traits. The proportion of single-seeded pods increased significantly, while that of three-seeded pods decreased significantly, with no significant change in two-seeded pods (Figure 3D). Meanwhile, the total seed number per plant of *tga9d-25* and *tga9d-26* was significantly lower than that of the wild type (WT), whereas no significant difference in 100-seed weight was observed compared to the WT (Figure S3).

**Figure 4:**
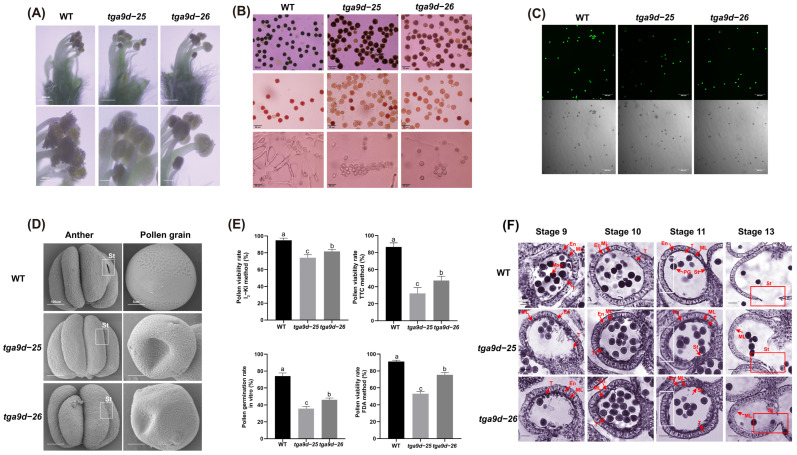
Phenotypic analysis of anthers and pollens of *tga9d* single mutant. (**A**) Analysis of pollen dispersal from anthers in WT, *tga9d-25* and *tga9d-26*. The scale bars from top to bottom are 500 μm and 200 μm. (**B**) Pollen viability analysis of WT, *tga9d-25* and *tga9d-26*. This panel shows results from three distinct viability assays. The top panels show I_2_-KI staining. The middle panels present 2,3,5-triphenyltetrazolium chloride (TTC) staining. The bottom panels depict results from the in vitro pollen germination assay. Bar = 50 μm. (**C**) Fluorescein diacetate (FDA) staining analysis of pollen viability in WT, *tga9d-25* and *tga9d-26*. Bar = 200 μm. (**D**) SEM analysis of dehiscent anthers and mature pollen grains. The white rectangle highlights the stomium (dehiscence slit) through which pollen is released. Scale bars from left to right are 100 μm and 5 μm. (**E**) Comparative statistical analysis of pollen viability in WT, *tga9d-25* and *tga9d-26* using I_2_-KI staining, TTC staining, in vitro pollen germination assays and FDA staining. Data are obtained from three biological replicates (*n* = 3), each represented by an independent individual plant. Three anthers per plant are analyzed as technical replicates. Three fields of view are randomly examined per anther to determine the pollen viability rate. Data are presented as the mean ± SD. Values with different letters indicate statistical differences (one-way ANOVA, Duncan’s test, *p*  <  0.05). (**F**) Histological analysis of cross-sections of anther locules from WT, *tga9d-25* and *tga9d-26* at different developmental stages, stained with hematoxylin. En, endodermis; ML, middle layer; T, tapetal layer; Msp, microspore; PG, pollen grain; St, septum. Bar = 20 μm.

To investigate how *GmTGA9d* affects the pod-setting efficiency in soybean, we conducted detailed research on the development of anthers and pollen grains in both mutants and WT plants during the flowering stage. We initially observed impaired pollen release from the anthers of opening flowers onto the stigmas in *tga9d-25* and *tga9d-26* compared to WT (Figure 4A). Then we used four different methods to assess pollen viability including I_2_-KI staining, 2,3,5-triphenyltetrazolium chloride (TTC) staining, fluorescein diacetate (FDA) staining and an in vitro pollen germination assay (Figure 4B,C). Consistent results from these four methods indicated reduced pollen viability in *tga9d-25* and *tga9d-26*, with particularly significant declines observed in both the in vitro pollen germination and TTC staining assays (Figure 4E). SEM observation further revealed that some pollen grains of *tga9d-25* and *tga9d-26* were malformed, displaying sunken or compressed shapes, indicating underlying anther structural defects (Figure 4D). These results suggested that the *GmTGA9d* mutation might have a more pronounced impact on the later stages of pollen maturation.

Then we examined the anther cross-sections of WT, *tga9d-25* and *tga9d-26* at different developmental stages of soybean anthers (Figure 4F). At Stage 10, the middle layer and tapetum were largely degraded in WT, but the two *tga9d* mutants exhibited delayed tapetum degeneration and abnormal thickening of the middle layer. At Stage 11, while these layers were completely degraded in the WT, the middle layer in the two *tga9d* mutants persisted and continued to thicken (Table S1). At Stage 13, the adjacent locules of WT had completely fused and undergone dehiscence. In contrast, in the two *tga9d* mutants’ anthers, remnants of the middle layer persisted along the inner walls of the locules. Although the locules had fused, anther dehiscence was abnormal, characterized by very short stomia, which severely compromised the efficiency of pollen release. SEM observations of dehisced anthers confirmed markedly smaller stomia in *tga9d* mutants, with some anthers displaying abnormal development in the adaxial locule (Figure 4D), a phenotype similar to the *Arabidopsis* *tga9tga10* double mutant. The results indicate that mutation of *GmTGA9d* causes persistent thickening of the anther middle layer, which in turn disrupts anther structure and ultimately hinders normal anther dehiscence and pollen release. Combined with the observed anther structural defects and plant phenotypes at maturity, such as altered podding traits and reduced total grain number per plant, we suggest that the reduction in seed quantity is influenced by multiple factors and the decreased pollen dispersal efficiency caused by abnormal anther dehiscence is most likely a crucial contributing factor.

In parallel, we created two *tga9c* mutants (*tga9c-8* and *tga9c-12*) (Figure S4A,B), which showed no detectable defects in pollen viability compared to WT (Figure S4C,D). The contrasting genetic evidence suggests that *GmTGA9d* may play a more critical role in regulating male fertility.

### 2.4. GmTGA9d Negatively Regulates GmDYT1c Expression

**Figure 5:**
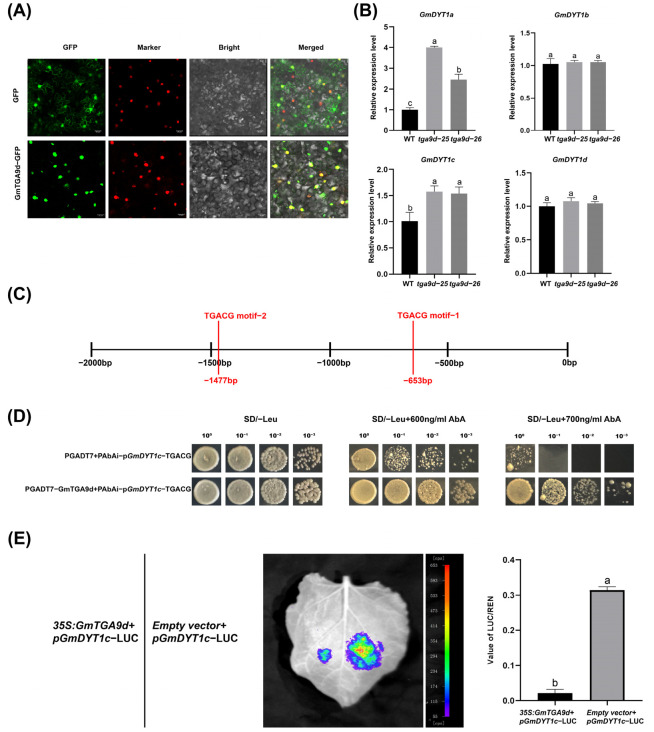
GmTGA9d binds to the *GmDYT1c* promoter and suppresses its expression. (**A**) Subcellular localization of GmTGA9d. Bar  =  20 μm. (**B**) Analysis of the relative expression levels of *GmDYT1a*, *GmDYT1b*, *GmDYT1c* and *GmDYT1d* in flowers of the WT, *tga9d-25* and *tga9d-26*. Data are obtained from three biological replicates (*n* = 3), and each biological replicate consists of pooled floral tissue samples from a single independent plant. For qRT-PCR analysis, each biological sample is examined in three technical replicates. The 2^−ΔΔCT^ method is used to calculate the relative expression levels. Data are presented as the mean ± SD. Values with different letters indicate statistical differences (one-way ANOVA, Duncan’s test, *p*  <  0.05). (**C**) Schematic diagram of the *GmDYT1c* promoter with two TGACG cis-elements within a 2000 bp region upstream of the ATG. (**D**) Yeast one-hybrid (Y1H) assay for the binding of GmTGA9d to the *GmDYT1c* promoter. Yeast cells from serial dilutions (1, 1:10, 1:100, and 1:1000) are plated on SD/−Leu/−Ura medium supplemented with 600 ng/mL and 700 ng/mL Aureobasidin A (AbA). The empty pGADT7 vector serves as the negative control. (**E**) Luciferase assays in tobacco leaves demonstrated that GmTGA9d suppresses the *GmDYT1c* promoter. Normalized luciferase activity (LUC/REN ratio) is compared between the experimental group (co-infiltrated with *35S::GmTGA9d* and *pGmDYT1c::LUC*) and the control group (co-infiltrated with empty vector and *pGmDYT1c::LUC*) to assess the effect of GmTGA9d on the transcriptional activity of the *GmDYT1c* promoter. Data are obtained from three independent biological replicates (*n* = 3), and each replicate is derived from distinct transiently transformed materials. For each biological replicate, leaves injected with the control and experimental combinations are collected simultaneously. For each treatment, three independent leaf discs (approximately 1 cm in diameter) are sampled as three technical replicates. The relative LUC/REN activity ratio is calculated for each leaf disc. Data are presented as the mean ± SD. Values with different letters indicate statistical differences (one-way ANOVA, Duncan’s test, *p*  <  0.05).

To elucidate the molecular function of *GmTGA9d*, we first determined its subcellular localization. The GmTGA9d protein was primarily localized to the nucleus, consistent with its predicted role as a transcription factor, with faint signals also detected in the cytoplasm (Figure 5A). Previous studies in *Arabidopsis* and rice have shown that *TGA9* and *TGA10* regulate anther tapetum development by directly controlling the expression of *DYT1* [37,38]. Based on this conserved regulatory module, we hypothesized that soybean *GmTGA9d* might function through a similar mechanism. To test this hypothesis, we identified four *DYT1* homologous genes in the soybean genome, designated *GmDYT1a*, *GmDYT1b*, *GmDYT1c* and *GmDYT1d*. Phylogenetic analysis revealed that GmDYT1a and GmDYT1d share higher similarity, as do GmDYT1b and GmDYT1c (Figure S5A). Multiple sequence alignment showed that the functional domains of GmDYT1a, GmDYT1b and GmDYT1c were highly conserved with those of DYT1 proteins from *Arabidopsis*, rice, maize and tomato (Figure S5B). In contrast, the amino acid sequence of GmDYT1d contained a truncation within its functional domain, suggesting that it may have lost its canonical function (Figure S5B). Subsequent subcellular localization analysis showed that GmDYT1b and GmDYT1c were localized in the cytoplasm, whereas GmDYT1a was localized in the nucleus, which suggested potential functional divergence among GmDYT1a, GmDYT1b and GmDYT1c (Figure S5C).

We then examined the expression levels of *GmDYT1a*, *GmDYT1b*, *GmDYT1c* and *GmDYT1d* in flowers from *tga9d-25*, *tga9d-26* and WT. Results showed that the expression of *GmDYT1a* and *GmDYT1c* was significantly upregulated in *tga9d-25* and *tga9d-26*, whereas the expression of *GmDYT1b* and *GmDYT1d* remained unchanged (Figure 5B). This suggested that GmTGA9d may transcriptionally suppress *GmDYT1a* and *GmDYT1c*. TGA transcription factors are known to function by specifically binding to the TGACG cis-element in target promoters [43]. Analysis of the promoters of the two upregulated genes revealed that the *GmDYT1c* promoter contains two TGACG cis-elements, whereas the *GmDYT1a* promoter lacks this motif, which suggested that GmTGA9d may indirectly regulate *GmDYT1a* (Figure 5C). Using a yeast one-hybrid assay, we found that the TGACG-1 element exhibited strong auto-activation activity. Therefore, we tested the remaining cis-element and found that GmTGA9d specifically binds to the TGACG-2 element in the *GmDYT1c* promoter (Figure 5D). A subsequent dual-luciferase reporter assay further demonstrated that GmTGA9d significantly suppressed the activity of the *GmDYT1c* promoter (Figure 5E). In conclusion, GmTGA9d, as a nucleus-localized transcription factor, directly binds to the TGACG element in the *GmDYT1c* promoter and suppresses its expression activity. These findings provided molecular evidence suggesting that *GmTGA9d* may participate in soybean anther development through the conserved TGA9-DYT1 regulatory module.

### 2.5. Genetic Evidence Supports Overlapping Roles of GmDYT1a and GmDYT1c in Soybean Male Fertility

**Figure 6:**
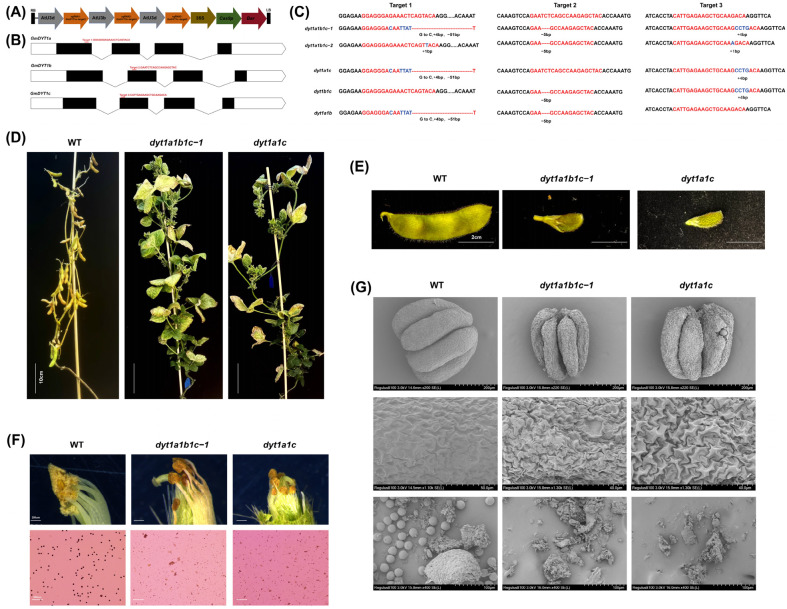
Generation and phenotypic characterization of *dyt1* multiple mutants. (**A**) Schematic diagram of the CRISPR-Cas9 gene-editing vector for *GmDYT1a/b/c*. (**B**) Schematic of the target sites in *GmDYT1a/b/c*. (**C**) Base editing status at the target sites in the five edited lines, *dyt1a1b1c-1, dyt1a1b1c-2, dyt1a1c, dyt1b1c* and *dyt1a1b*. The target sequences are shown in red text. (**D**) Comparison of mature plants of WT, *dyt1a1b1c-1* and *dyt1a1c*. Bar  =  10 cm. (**E**) Comparison of mature pods of the WT, *dyt1a1b1c-1* and *dyt1a1c*. Bar  =  2 cm. (**F**) Comparative analysis of anthers and pollen grains from the WT, *dyt1a1b1c-1* and *dyt1a1c*. The three upper panels show microscopic observation of pollen dispersal from anthers of opening flowers. Bar  =  250 μm. The three lower panels present I_2_-KI staining analysis of pollen viability. Bar  =  200 μm. (**G**) Comparative SEM analysis of anthers and pollen grains from the WT, *dyt1a1b1c-1* and *dyt1a1c.* The upper row compares anther morphology among WT, *dyt1a1b1c-1* and *dyt1a1c*. Bar = 200 μm. The central row compares the anther surface of WT, *dyt1a1b1c-1* and *dyt1a1c*. The scale bars from left to right are 50 μm, 40 μm, and 40 μm. The lower row compares the pollen release from crushed anthers of WT, *dyt1a1b1c-1* and *dyt1a1c*. Bar  =  100 μm.

To investigate the function of *GmDYT1* genes in soybean male fertility, we simultaneously edited *GmDYT1a*, *GmDYT1b* and *GmDYT1c* by CRISPR-Cas9 technology (Figure 6A,B) and obtained a T_0_-generation-positive plant designated as *dyt1-21*. Sequencing confirmed that the plant contained heterozygous mutations at all three target sites (Figure S6A). We found that *dyt1-21* exhibited normal pollen fertility (Figure S6C). By successively selfing *dyt1-21* to the T_3_ generation, we isolated and obtained four types of homozygous mutants, including two *dyt1a1b1c* triple mutants with different editing types and one editing type of the *dyt1a1c*, *dyt1a1b* and *dyt1b1c* double mutants (Figure 6C). Sequencing analysis verified that these edits caused premature translation termination within the functional domain, resulting in loss of function (Figure S6A,B). Phenotypic analysis revealed that the two *dyt1a1b1c* mutants and the *dyt1a1c* mutant displayed fully normal vegetative growth and maintained vigorous vegetative development throughout the late reproductive stage, and produced a large number of underdeveloped pods, which represents a common characteristic of complete male-sterile materials (Figure 6D,E). Detailed observation of the floral organs of *dyt1a1b1c-1* and *dyt1a1c* showed that their anthers failed to dehisce, appearing shrunken and wrinkled. I_2_-KI staining confirmed the complete absence of stainable pollen grains within the anthers of *dyt1a1b1c-1, dyt1a1b1c-2* and *dyt1a1c* (Figure 6F and Figure S6C). In contrast, pollen observation of *dyt1a1b* and *dyt1b1c* revealed completely normal fertility (Figure S6C). SEM observation further revealed that the anthers of *dyt1a1b1c-1* and *dyt1a1c* were significantly smaller, shrunken and covered with furrows compared to the plump, smooth anthers of the WT. When anthers were mechanically crushed, WT anthers released abundant pollen grains, while only anther fragments and no pollen grains were observed in *dyt1a1b1c-1* and *dyt1a1c* (Figure 6G). These results provide genetic evidence that *GmDYT1a* and *GmDYT1c* have overlapping roles in soybean anther development, and their simultaneous mutation is associated with complete male sterility.

### 2.6. Simultaneous Mutation of GmDYT1a and GmDYT1c Is Associated with Abnormal Anther Development

**Figure 7:**
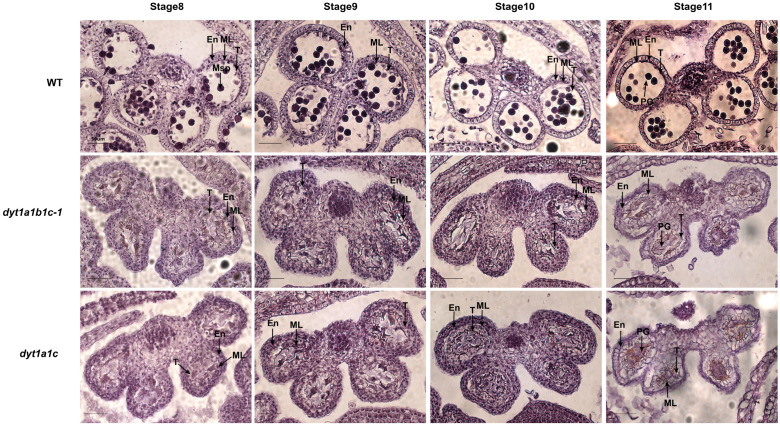
Comparative analysis of hematoxylin-stained cross-sections of anthers at different developmental stages in the WT, *dyt1a1b1c-1* and *dyt1a1c*. En, endodermis; ML, middle layer; T, tapetal layer; Msp, microspore; PG, pollen grain. Bar = 50 μm.

To elucidate the cytological cause and identify the critical stage of pollen abortion in the male-sterile *dyt1a1b1c* and *dyt1a1c* mutants, we performed comparative observations of paraffin sections from anthers at Stage 8 to Stage 11 in both mutants and WT (Figure 7). Results showed that the *dyt1a1b1c* and *dyt1a1c* mutants exhibited severe anther developmental defects at Stage 8. Their anther walls were significantly thickened, and the abnormally enlarged middle layer and tapetum cells occupied most of the locule space, severely compressing the locule structure. No normal microspore cells could be observed within the locules, suggesting that the abortion process initiated at an early developmental stage. At Stage 11, pollen grains in WT anthers were fully developed, whereas the locule structure in *dyt1a1b1c* and *dyt1a1c* mutants had completely collapsed and appeared highly irregular. The persistently enlarged and undegraded middle layer and tapetum tissues remained, and the few residual pollen grains were compressed into irregular shapes, ultimately resulting in complete pollen abortion. Overall, the simultaneous mutation of *GmDYT1a* and *GmDYT1c* was associated with severe disruption of anther wall development, which may contribute to complete male sterility.

## 3. Discussion

Soybean is a crucial crop for food, feed and oil, and improving its yield and quality is vital for global food security. Although the “three-line” hybrid breeding system based on cytoplasmic male sterility has achieved progress, research into developing new hybrid breeding methods utilizing nuclear male sterility (NMS) in soybean remains limited [44]. The establishment of a new hybrid breeding system requires systematic identification and functional studies of NMS genes that regulate male fertility [45]. Normal anther development is essential for male fertility, and the patterned formation of its somatic cell layers is a strictly regulated genetic process. At the apex of the stamen primordium, archesporial cells undergo periclinal division, forming outer primary parietal cells and inner primary sporogenous cells. The primary parietal cells further divide and differentiate, eventually developing into the four somatic cell layers of the mature anther, including the epidermis, endothecium, middle layer and tapetum [46,47]. In *Arabidopsis*, rice and maize, bZIP transcription factors like TGA9/10 and ROXY-type glutaredoxins play a central role in initiating archesporial cell differentiation and early somatic cell fate determination [20,48,49]. Subsequently, signaling mediated by the TPD1-EMS1 module precisely regulates the proliferation of archesporial cells, thereby initiating the differentiation of the four somatic wall layers [50,51]. Furthermore, a network involving bHLH transcription factors such as DYT1, bHLH010, bHLH089 and bHLH090 controls tapetum differentiation, function and programmed degradation [52]. These regulatory modules are highly conserved across different species. However, there remains a significant gap in research on soybean.

To explore the regulatory function of TGA9 in soybean anther development and male fertility, we systematically identified four *GmTGA9* genes in soybean. CRISPR/Cas9 technology was used to simultaneously edit multiple members of the *GmTGA9* gene family [53]. Phenotypic analysis of T_0_ plants with different *GmTGA9* editing combinations revealed an obvious male-sterile phenotype, which occurred in plants involving both *GmTGA9c* and *GmTGA9d*. This indicated that these two genes might be involved in the regulation of male fertility in soybean (Figure 2D–F). We further generated single-gene homozygous mutants of *tga9c* and *tga9d*. The *tga9d* single mutant exhibited partial male sterility, while the *tga9c* single mutant showed no obvious developmental defects (Figure 3C and Figure S4C,D). Cytological evidence demonstrated that the loss of *GmTGA9d* function resulted in abnormal degradation of the anther middle layer. This defect led to incomplete anther dehiscence, reduced pollen release efficiency and decreased pollen viability, which may serve as a key reason for the significant increase in the proportion of single-seeded pods in mutant plants at the mature stage (Figure 4B–F). The male-sterile defects observed in *tga9d* mutants were highly consistent with those of TGA transcription factor mutants in *Arabidopsis*, rice and maize [14,37,38]. These findings suggest that the TGA-mediated regulatory pathway is highly conserved among higher plants, and soybean shares a similar regulatory mechanism for male fertility (Figure 1C,D).

In *Arabidopsis* and rice, TGA9/10 transcription factors directly regulated *DYT1* expression, thereby initiating the transcriptional pathway for anther development and function [35,36]. This study supports the conservation of this core regulatory pathway in soybean, as we demonstrated that GmTGA9d directly bound to the *GmDYT1c* promoter and suppressed its transcription (Figure 5B–E). Meanwhile, we found that the expression of *GmDYT1a* was significantly upregulated in the *tga9d* mutant. Since the *GmDYT1a* promoter lacks the TGACG elements, this regulation is likely to be indirect. This indirect regulatory effect might be due to a compensatory feedback response triggered by the release of direct repression of *GmDYT1c* by GmTGA9d, or it could occur via a transcriptional cascade downstream of GmTGA9d. We then found that genetic evidence supports overlapping roles of *GmDYT1a* and *GmDYT1c* in regulating soybean male fertility, as the simultaneous mutation of both genes was associated with male sterility (Figure 6D–F). In contrast, only a single *DYT1* gene exists in *Arabidopsis*, rice, and maize. This highlights the functional uniqueness of DYT1 in soybean and plays crucial roles in its anther development process.

Subcellular localization analysis revealed a divergence. GmDYT1a was localized to the nucleus, whereas GmDYT1c was found primarily in the cytoplasm (Figure S5C). This distribution differed from the typical nuclear localization of bHLH transcription factors. Based on this differential localization, we speculated that the nuclear-localized GmDYT1a likely functions in transcriptional regulation. In contrast, the cytoplasmic GmDYT1c may play roles through distinct mechanisms. For example, it might require specific signals or modifications for nuclear import to regulate transcription. Alternatively, it might be involved in non-transcriptional processes within the cytoplasm, such as signal transduction or modulating protein interaction networks. The precise molecular mechanisms underlying these potential functions await further experimental investigation.

Based on comprehensive cytological analysis integrating molecular and genetic evidence, we found that the anther wall underwent a tightly regulated spatiotemporal degradation pattern during development. Both the partial sterility of the *tga9d* single mutant and the complete sterility of the *dyt1a1c* double mutant ultimately pointed to defects in the middle layer and tapetum, although the timing and severity of these defects were markedly different. In the two *tga9d* mutants, delayed degradation and thickening of the middle layer were observed (Figure 4F). This developmental defect physically interfered with anther dehiscence, reducing pollen release efficiency, and likely subtly compromised the supportive function of the tapetum for pollen maturation. Moreover, the *dyt1a1c* double mutant exhibited abnormal enlargement of the middle layer and the tapetum cells and a complete failure of the PCD program as early as the pre-meiotic stage (Figure 7). This severely disrupted the microenvironment for microsporogenesis, leading to a complete block of pollen development. Our findings underscore that anther wall development is not a simple event but rather a continuous, spatiotemporally ordered process governed by a precise genetic network. Mutations in any gene within this pathway may disrupt the development of the anther wall layers, ultimately resulting in male sterility phenotypes of varying severity.

In conclusion, this study establishes a crucial theoretical foundation for the future construction and optimization of a soybean hybrid breeding system based on NMS.

## 4. Materials and Methods

### 4.1. Plant Materials and Growth Conditions

Soybean cultivar *Williams 82* (W82) and all CRISPR/Cas9 transgenic plants were grown in the greenhouse of Nanjing Agricultural University with a 12 h light/12 h dark photoperiod and a temperature of 28  °C/22  °C (day/night). *Nicotiana benthamiana* was grown under conditions of 16 h light/8 h dark in an illuminated incubator (RXZ-430D, Ningbo Jiangnan, Ningbo, China) at 23 °C and was used for subcellular localization and dual-luciferase (LUC) assays.

### 4.2. Construction of Phylogenetic Trees and Sequence Alignment

The protein sequences of GmTGA9a, GmTGA9b, GmTGA9c and GmTGA9d, as well as TGA9 homologous sequences from other species—including wild soybean (*Glycine soja*), cowpea (*Vigna unguiculata*), maize (*Zea mays*), rice (*Oryza sativa*), *Arabidopsis* (*Arabidopsis thaliana*), sorghum (*Sorghum bicolor*) and cacao (*Theobroma cacao*)—were obtained from the Phytozome database. Similarly, the sequences of GmDYT1a, GmDYT1b, GmDYT1c and the DYT1 homologs from maize, rice, *Arabidopsis* and tomato (*Solanum lycopersicum*) were downloaded from Phytozome [54]. Based on these sequences, the phylogenetic tree was constructed using MEGA 11.0 software by the neighbor-joining method with 1000 bootstrap replicates. Multiple sequence alignments were performed using the ESPript 3.0 online server [55].

### 4.3. Construction of CRISPR/Cas9 Vector and Soybean Transformation

The CRISPR-Cas9 gene-editing vectors used in this study included AtU3b, AtU3d and pYLCRISPR/Cas9P35S-B. Vector construction was performed strictly following the manufacturer’s instructions [56]. Target sequences for the *GmTGA9* and *GmDYT1* genes were designed using the online tool CRISPR-GE (http://skl.scau.edu.cn/, accessed on 10 April 2026) [57]. The successfully constructed recombinant vectors were then introduced into *Agrobacterium tumefaciens* strain EHA105 competent cells. Finally, genetic transformation of the soybean recipient cultivar W82 was performed using the soybean hypocotyl node transformation method [58].

### 4.4. Identification of CRISPR/Cas9-Edited Transgenic Lines

To identify transgenic events, the DNA samples were first amplified using vector-specific primers targeting the T-DNA region (Supplementary Table S2). Samples yielding the expected 2046 bp amplicon were considered PCR-positive for the transgene. For these positive plants, the genomic regions flanking the target sites were then amplified and sequenced by Sanger sequencing to determine the editing profiles. The primer sequences used for sequencing are listed in Supplementary Table S2. Putative mutants with the desired editing patterns were allowed to self-pollinate, and their progeny were sequenced to confirm the heritability and stability of the mutations.

### 4.5. Subcellular Localization Assay

The full-length coding sequences (CDSs) of *GmTGA9d*, *GmDYT1a*, *GmDYT1b*, and *GmDYT1c* (stop codons excluded) were amplified from W82 flower cDNA. Each fragment was then inserted into the BglII-linearized pCAMBIA3301-GFP vector using a one-step cloning kit (Vazyme, Nanjing, China) to generate the respective *p35S::GmTGA9d*-GFP, *p35S::GmDYT1a*-GFP, *p35S::GmDYT1b*-GFP, and *p35S::GmDYT1c*-GFP fusion constructs. For transient expression, leaves of *Nicotiana benthamiana* were infiltrated with *Agrobacterium tumefaciens* harboring either the recombinant vectors or the empty pCAMBIA3301-GFP control vector. The infiltrated leaves were then incubated for 2 days at room temperature before imaging. GFP fluorescence was detected using a Zeiss LSM780 (Carl Zeiss AG, Oberkochen, Germany) confocal laser scanning microscope with a 488 nm excitation wavelength.

### 4.6. Pollen Viability Assay

Pollen viability was comprehensively assessed using multiple methods, including iodine-potassium iodide (I_2_-KI) staining, 2,3,5-triphenyltetrazolium chloride (TTC) staining, fluorescein diacetate (FDA) staining, anther dehiscence observation and in vitro pollen germination assays. For all staining assays, anthers were collected from mature flower buds expected to open the next day and were carefully excised and processed according to the respective reagent protocols, and then observed and calculated under an Olympus CX31 microscope (Tokyo, Japan) [3]. For I_2_-KI and TTC staining, three random microscopic fields were selected for each sample, with no fewer than 200 pollen grains counted per field. Pollen grains stained dark blue-black by I_2_-KI or red by TTC were defined as viable. Pollen viability (%) = (number of viable pollen grains/total number of observed pollen grains) × 100%. FDA fluorescence signals were visualized and captured using a Zeiss LSM780 confocal laser scanning microscope. At least three random fields of view were photographed for each sample. Pollen grains showing bright green fluorescence were regarded as viable. Pollen viability (%) = (number of viable pollen grains/total number of observed pollen grains) × 100%. Observations of anther dehiscence and in vitro pollen germination were conducted between 8:00 and 10:00 AM using randomly selected flowers that had just opened on the same day. Anther dehiscence was directly examined under a stereomicroscope. For in vitro germination, pollen grains were collected from freshly opened flowers and evenly inoculated onto pollen germination medium (PGM) plates. Three random microscopic fields were selected on each plate, with no fewer than 100 pollen grains counted per field. Pollen grains with a pollen tube length exceeding the pollen grain diameter were considered germinated. Pollen germination rate (%) = (number of germinated pollen grains/total number of observed pollen grains) × 100%. The PGM was prepared by dissolving 15% (*w*/*v*) sucrose, 0.03% (*w*/*v*) Ca(NO_3_)_2_, 0.04% (*w*/*v*) H_3_BO_3_, and 0.6% (*w*/*v*) agar in deionized water, boiling the mixture for 10 min, and then pouring it into culture dishes [59]. The plates were then immediately placed in a 30 °C incubator in the dark for 30 min before germination rates were calculated under an Olympus CX31 microscope (Tokyo, Japan).

### 4.7. Scanning Electron Microscopy (SEM) Observation

For SEM observation, anthers at Stage 10 and Stage 12 were fixed in 3% glutaraldehyde at 4 °C, dehydrated through a graded series of ethanol (30%, 50%, 70%, 80%, 90%, and 100%; 5 min per step), and critical-point-dried. Samples were then sputter-coated with gold and imaged using a HITACHI Regulus 8100 scanning electron microscope (Hitachi High-Technologies Corporation, Tokyo, Japan).

### 4.8. Paraffin Section Assay

With reference to the classification criteria for anther developmental stages in rice [38], soybean anther development was divided from Stage 8 to Stage 13 according to different flower bud lengths (Table S3). Flower buds at different developmental stages from mutants and the wild type (WT) were collected and fixed in FAA solution (5% formaldehyde, 10% glacial acetic acid, 50% ethanol). Before fixation, samples were subjected to vacuum infiltration for 10 min to ensure complete immersion and settling at the bottom of the tube. Subsequently, the tissues were dehydrated through a graded ethanol series at room temperature: 30%, 50%, 70%, 85%, 95%, and 100% ethanol, 30 min per step. Following dehydration, clearing was performed by sequential incubation in 100% ethanol, an ethanol/xylene mixture (1:1, *v*/*v*), and 100% xylene, 60 min per step. The samples were then transferred to a 60 °C oven and processed sequentially as follows: a paraffin/xylene mixture (1:1, *v*/*v*, 60 min per step), followed by two changes of pure paraffin (60 min per step). Subsequently, the tissues were infiltrated with pure paraffin for 3–5 days. After infiltration, the samples were embedded in pure paraffin. Following block trimming, sections were cut using a microtome (Leica Microsystems GmbH, Wetzlar, Germany) and stained with hematoxylin, and then observed and photographed using an Olympus CX31 microscope (Tokyo, Japan).

### 4.9. Yeast One-Hybrid (Y1H) Assay

To construct the effector vector for the yeast one-hybrid assay, the full-length CDS of *GmTGA9d* (without the stop codon) was amplified from its GFP fusion vector and ligated into the pGADT7 plasmid to generate the effector vector pGADT7-*GmTGA9d*. For the reporter vectors, DNA fragments containing TGACG-1 and TGACG-2 elements from the *GmDYT1c* promoter region were separately cloned into the pAbAi plasmid to generate the reporter vectors pAbAi-p*GmDYT1c*-TGACG-1 and pAbAi-p*GmDYT1c*-TGACG-2. The assay was performed according to the manufacturer’s (Clontech, Palo Alto, CA, USA) instructions. First, the reporter vectors pAbAi-*pGmDYT1c*-TGACG-1/2 were individually introduced into Y1H Gold yeast competent cells. Positive transformants were screened on SD/-Ura medium supplemented with Aureobasidin A (AbA) at an appropriate concentration to select positive transformants and test for auto-activation. Subsequently, the effector vector pGADT7-*GmTGA9d* or negative control vectors were separately transformed into the above bait–reporter yeast strains. The transformed yeast cells were plated on SD/-Ura/-Leu/AbA dropout medium to evaluate the interaction between GmTGA9d and the TGACG elements in the *GmDYT1c* promoter.

### 4.10. LUC Reporter Assay

To construct the vector for the LUC reporter assay, the full-length CDS of *GmTGA9d* was amplified from its GFP fusion vector and inserted into the BamHI-linearized pCAMBIA3301-26 vector using a one-step cloning kit (Vazyme, Nanjing, China) to generate the effector construct *p35S::GmTGA9d*. This effector plasmid was then transformed into *Agrobacterium tumefaciens* strain GV3101 for subsequent agroinfiltration. The reporter vector was constructed by cloning the promoter sequence of *GmDYT1c* into the pGreenII 0800-LUC vector. This reporter construct and the helper plasmid pSoup19 were co-transformed into *Agrobacterium* GV3101. The empty pCAMBIA3301-26 vector served as the negative control. The reporter strain was then mixed with an equal volume of either the effector (*p35S::GmTGA9d*) or control (pCAMBIA3301-26) strain for co-infiltration. The mixed bacterial suspensions were co-infiltrated into leaves of *Nicotiana benthamiana.* After incubation for 2–3 days, leaf samples were harvested, and firefly and Renilla luciferase activities were measured using the Dual-Luciferase Reporter Assay System (Promega Corporation, Madison, WI, USA), and LUC/REN ratios were calculated.

### 4.11. qRT-PCR Analysis

Total RNA was extracted from WT and the *tga9d* mutants using TRIzol reagent (Accurate Biology, Changsha, China). After quality assessment, the RNA was reverse-transcribed into cDNA using the Evo M-MLV RT Mix Kit with gDNA Clean for qPCR (Ver. 2) (Accurate Biology, China). A Bio-Rad CFX96 real-time system was applied to run the qRT-PCR assay with SYBR qPCR Master Mix (Accurate Biology, China). Specific primers for all tested genes (*GmTGA9a, GmTGA9b, GmTGA9c, GmTGA9d, GmDYT1a, GmDYT1b, GmDYT1c* and *GmDYT1d*) were designed (Table S2). To ensure primer specificity, especially for highly homologous gene family members, the following validation procedures were performed: (i) Conventional PCR amplification and agarose gel electrophoresis were conducted to confirm that each primer pair yielded only a single band with the correct fragment size. (ii) Melting curve analysis was carried out after qPCR to verify single-peak signals, identifying non-specific amplification and primer dimer formation.

All experiments were carried out with three biological replicates and three technical replicates. The 2^−ΔΔCT^ method was used to calculate the relative expression levels with the *GmTubulin* serving as the internal reference [60].

### 4.12. Statistical Analysis

Comparisons between two experimental groups were conducted using Student’s *t*-test (*p* < 0.05). For comparisons involving more than two groups, one-way ANOVA was applied followed by Duncan’s multiple comparison test (*p* < 0.05).

## 5. Conclusions

This study revealed the function of a conserved GmTGA9-GmDYT1 module in regulating anther wall development and male fertility in soybean. The results demonstrated that the transcription factor GmTGA9d modulated anther development by directly repressing the expression of its downstream target gene *GmDYT1c*. Loss of *GmTGA9d* function triggered delayed degradation and abnormal thickening of the anther middle layer, thereby causing abnormal anther dehiscence and ultimately resulting in partial male sterility. Further genetic analyses provided evidence that *GmDYT1a* and *GmDYT1c* had overlapping roles in soybean male fertility. Simultaneous mutation of these two genes was associated with severe developmental defects in the tapetum and middle layer at early developmental stages, ultimately leading to complete male sterility. These findings not only advance our understanding of the regulatory pathway underlying soybean anther wall development, but also provide a molecular foundation for future breeding improvement by utilizing the relevant regulatory mechanisms.

## Figures and Tables

**Table 1:** Summary of genotypes and male fertility of T_0_ heterozygous mutants of *GmTGA9*.

Mutant	*GmTGA9a*	*GmTGA9b*	*GmTGA9c*	*GmTGA9d*	Male Fertility
*tga9-8*	Het	Het	Het	Het	Sterile
*tga9-11*	Het	WT	Het	Het	Sterile
*tga9-33*	WT	Het	WT	Het	Fertile
*tga9-39*	WT	WT	Het	Het	Sterile
*tga9-59*	Het	WT	WT	Het	Fertile

**Note:** “Het” indicates heterozygous editing at the corresponding gene target site, while “WT” represents no editing at this target site.

## Data Availability

The original contributions presented in this study are included in the article/Supplementary Materials. Further inquiries can be directed to the corresponding authors.

## Supplementary Materials

The following supporting information can be downloaded at https://www.mdpi.com/article/10.3390/plants15101510/s1. Figure S1. Comparison of target-site sequencing chromatograms and phenotypes of *tga9-59* and *tga9-33*. (A) Comparison of mature pollen viability by I_2_-KI staining among wild type (WT), T_0_ progeny of *tga9-59*, and T_0_ progeny of *tga9-33*. Bar = 200 μm. (B) Analysis of editing events and sequencing chromatograms at the target sites of *GmTGA9a*, *GmTGA9b*, *GmTGA9c* and Gm*TGA9d* in the T_2_ progeny of *tga9-59*. (C) Analysis of editing events and sequencing chromatograms at the target sites of *GmTGA9a*, *GmTGA9b*, *GmTGA9c* and Gm*TGA9d* in the T_2_ progeny of *tga9-33*. The target sequences are marked in black text above the chromatograms; the schematic to the right depicts the predicted effects of these mutations on the protein domains. (D) Comparison of mature pollen viability by I_2_-KI staining among the WT, T_2_ progeny of *tga9-59* and T_2_ progeny of *tga9-33*. Bar = 200 μm. Figure S2. Analysis of editing events at the target sites in *tga9d-25* and *tga9d-26*. (A) Analysis of editing events and sequencing chromatograms at the target sites in *tga9d-25* and *tga9d-26*. (B) Comparison of the amino acid sequences resulting from the edits at the target sites in *tga9d-25* and *tga9d-26*. Figure S3. Statistical analysis of seed number per plant and 100-seed weight in WT, *tga9d-25* and *tga9d-26* at maturity. Data are obtained from eight biological replicates (*n* = 8), with statistics collected from eight independently grown plants. Data are presented as the mean ± standard deviation (SD). Values with different letters indicate statistical differences (one-way ANOVA, Duncan’s test, *p*  <  0.05). Figure S4. Generation and phenotypic analysis of the two *tga9c* mutants. (A) Schematic diagram of locations of the two target sites within the *GmTGA9c* gene sequence and the CRISPR-Cas9 vector for *GmTGA9c* single-gene editing. (B) Base editing status at the target sites in the two edited lines, *tga9c-8* and *tga9c-11*. The target sequences are shown in red text. Editing events and sequencing chromatograms at the target sites are shown below the sequence, and the schematic to the right depicts the predicted impact of the edits on the protein domains. (C) Pollen viability analysis of WT, *tga9c-8* and *tga9c-11* by I_2_-KI staining and TTC staining. Bar = 50 μm. (D) Pollen viability analysis of WT, *tga9c-8* and *tga9c-11* by in vitro pollen germination assay and scanning electron microscopy (SEM) observation. Scale bars from upper to lower are 50 μm and 5 μm. Figure S5. Identification of *DYT1* homologous genes in soybean. (A) Phylogenetic tree analysis of DYT1 in multiple plant species, including *Arabidopsis thaliana*, *Solanum lycopersicum*, *Zea mays*, *Oryza sativa* and *Glycine max*. (B) Protein sequence alignment of four *GmDYT1* genes and their homologous genes in *Arabidopsis thaliana*, *Oryza sativa* and *Zea mays*. Black-box region indicates the conserved bHLH domain. (C) Subcellular localization of GmDYT1a, GmDYT1b and GmDYT1c. Bar = 20 μm. Figure S6. Analysis of editing events at the target sites and phenotypic analysis in different types of *dyt1* mutants. (A) Comparison of editing events and sequencing chromatograms at the target sites among *dyt1-21*, *dyt1a1b1c-1*, *dyt1a1b1c-2*, *dyt1a1c*, *dyt1b1c* and *dyt1a1b*. The blue highlighted region indicates the target sequence. (B) Comparison of the amino acid sequences resulting from the edits at the target sites in *dyt1a1b1c-1*, *dyt1a1b1c-2*, *dyt1a1c*, *dyt1b1c* and *dyt1a1b*. Black-box region indicates the bHLH domain. (C) Pollen viability analysis of *dyt1-21*, *dyt1a1b1c-2*, *dyt1b1c* and *dyt1a1b* by I_2_-KI staining. Bar = 200 μm. Table S1. Comparisons of middle layer thickness in transverse sections of anthers at the Stage 11 among WT, *tga9d-25* and *tga9d-26*. Data are obtained from three biological replicates (*n* = 3), with statistics collected from three independent anther transverse sections. For each anther transverse section, three different anther locules are selected for determination, serving as three technical replicates. Data are presented as the mean ± standard deviation (SD). Values with different letters indicate statistical differences (one-way ANOVA, Duncan’s test, *p* < 0.05). Table S2. Primers used in this study. Table S3. Classification of soybean anther developmental stages.
