# Supplementary figures and images for: GmTGA9-GmDYT1 Regulates Anther Wall Development to Affect Male Fertility in Soybean
Source: Plants (Basel). 2026 May 15;15(10):1510. doi: 10.3390/plants15101510 (PMC13210509; doi:10.3390/plants15101510)

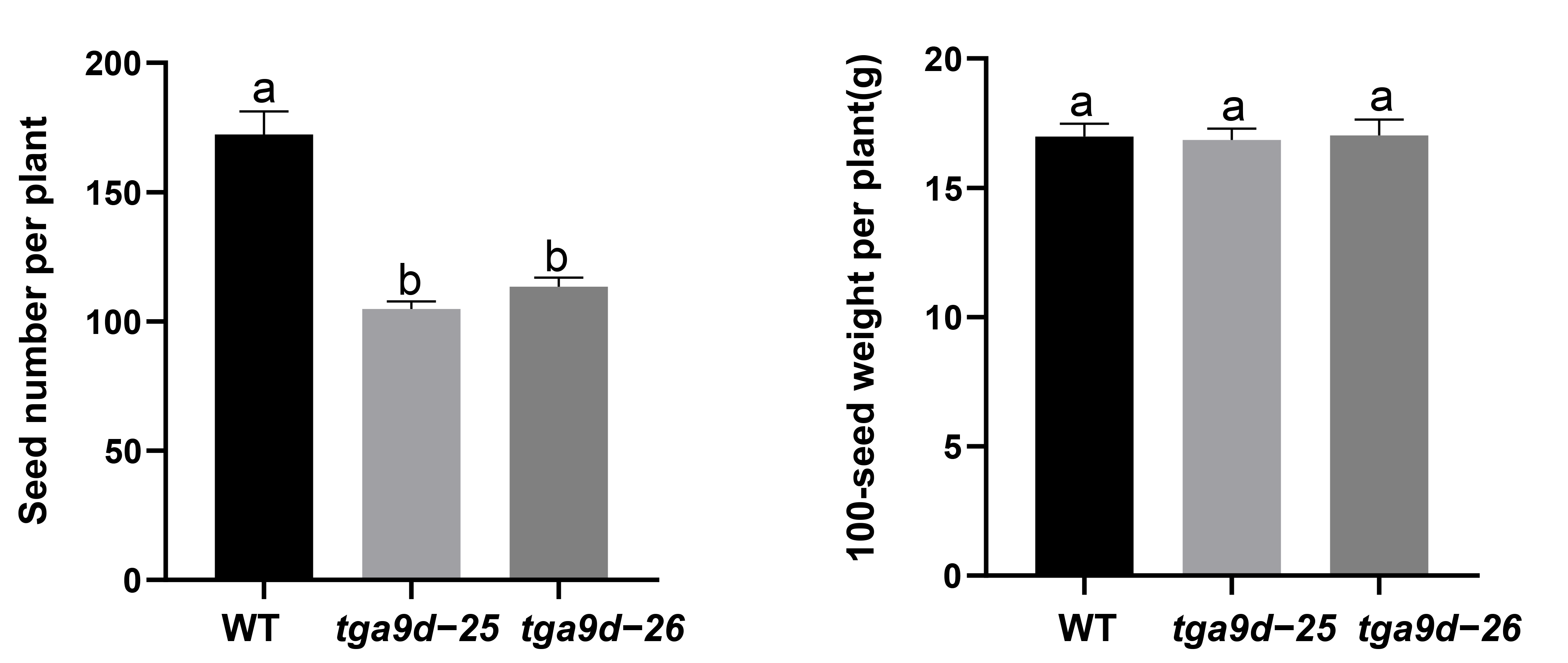

Supplement: Supplementary file 1 [file plants-15-01510-s001.zip › Figure S3.tif]
